# BHX, a novel pyrazoline derivative, inhibits breast cancer cell invasion by reversing the epithelial-mesenchymal transition and down-regulating Wnt/β-catenin signalling

**DOI:** 10.1038/s41598-017-09655-7

**Published:** 2017-08-22

**Authors:** Hanmei Bao, Qing Zhang, Zhongling Zhu, Hui Xu, Fengxia Ding, Meisa Wang, Shuangshuang Du, Yibo Du, Zhao Yan

**Affiliations:** 1 Department of Clinical Pharmacology, Tianjin Medical University Cancer Institute and Hospital, Tianjin, 300060 China; 2 Key Laboratory of Cancer Prevention and Therapy, Tianjin, Tianjin’s Clinical Research Center for Cancer, National Clinical Research Center for Cancer, Tianjin Medical University Cancer Institute and Hospital, Tianjin, 300060 China; 3 Department of Hematology, Tianjin Medical University Cancer Institute and Hospital, Tianjin, 300060 China

## Abstract

The novel pyrazoline derivative, BHX, has recently been shown to exhibit potent anti-tumour activity by blocking the Wnt/β-catenin signalling pathway. However, its effect on breast cancer growth and invasion are unknown. Our results show that BHX suppresses MDA-MB-231 cell viability and colony formation in a dose-dependent manner, and induces apoptosis and G0/G1 phase arrest. BHX-treated breast cancer cells showed morphological characteristics of cells undergoing apoptosis. Furthermore, BHX inhibited cell migration and invasion, which was associated with increased E-cadherin mRNA and protein expression, and down-regulation of *SNAIL* and vimentin. In addition, BHX induced the generation of intracellular ROS and decreased β-catenin protein and mRNA expression. We used a mouse xenograft model to investigate the effects of BHX *in vivo*, where the growth of MDA-MB-231 xenografted tumours was suppressed in nude mice treated continuously with BHX for 21 days. Finally, the rat plasma concentration of BHX was measured by ultra-performance liquid-chromatography tandem mass spectrometry and the pharmacokinetic parameters of BHX were processed by non-compartmental analysis. In conclusion, BHX merits further study as a novel therapeutic small molecule for the treatment of breast cancer.

## Introduction

Breast cancer is a common malignancy and ranks as the fourth leading cause of cancer mortality in women worldwide, with approximately 1.7 million new cases diagnosed every year^1^. Despite recent major advances in the understanding of the mechanisms of breast cancer progression and in the development of novel therapeutic modalities, mortality rates remain high. In the United States, the 5-year survival rate is only 23% for patients diagnosed with distant metastasis^2^. Tumour metastasis remains the most critical factor influencing the effectiveness of breast cancer therapy^3^. Therefore, suppressing proliferation and inhibiting breast cancer cell migration is considered to be an effective therapeutic strategy.

Deregulation of Wnt/β-catenin signalling plays an important role in cancer development and progression^4^. β-catenin is normally kept inactive by a complex composed of Axis inhibitor, Adenomatous polyposis coli, Casein kinase 1, and Glycogen-synthase kinase 3β^5^. Upon Wnt activation, GSK-3β is relieved of its ability to phosphorylate and destabilize β-catenin. This results in the accumulation of β-catenin in the cytoplasm and its subsequent translocation to the nucleus. Nuclear β-catenin forms a complex with other transcription factors of the T-cell factor/lymphocyte-enhancer-binding factor family to activate transcription of downstream target genes, such as CD44, cyclin D1, c-myc, Survivin, and Trib2^6^. Canonical activation of Wnt/β-catenin signalling is associated with poor prognosis in breast cancer patients^7^. Preclinical studies have shown that Wnt10b-driven activation of β-catenin stimulates the proliferation of metastatic breast cancer cells^8^.

Metastasis, considered the primary cause of mortality in most cancer patients, is a multi-step process that involves the detachment of tumour cells from primary sites, cell migration via systemic circulation, extravasation to secondary sites, and further proliferation the metastatic cells to form new tumours^9^. The epithelial-mesenchymal transition (EMT) is a developmental process in which epithelial cells lose polarity and acquire migratory and invasive properties, thereby becoming mesenchymal-like cells^10^. EMT has been shown to play a critical role during the initiation of metastasis^11^. Cancer cells that undergo EMT lose epithelial marker expression, like E-cadherin, and express mesenchymal markers, like vimentin, α- smooth muscle actin, and N-cadherin^12^. EMT markers are frequently expressed in various types of breast cancers, suggesting a strong association between the EMT program and breast cancer progression^13^. Additionally, EMT is associated with an enhancement of cell motility, and an increase in the expression and activity of matrix metalloproteinases^14^.

**Figure 1:**
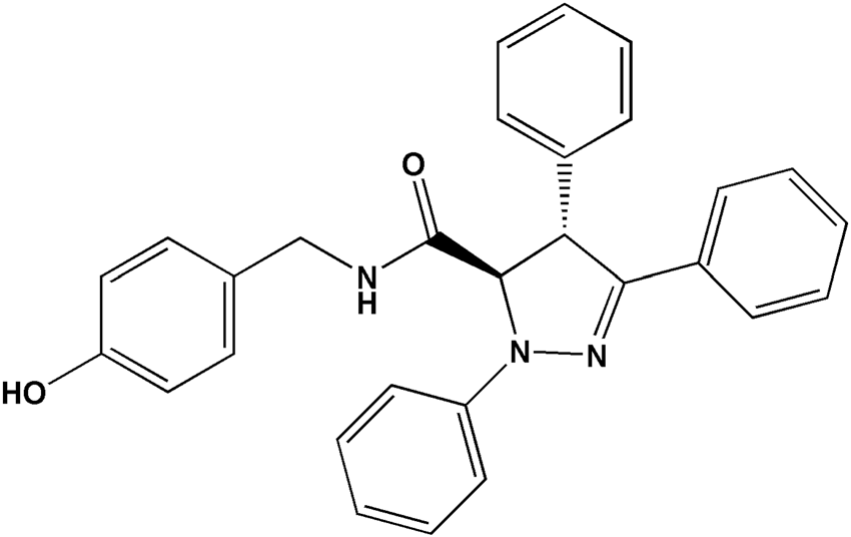
Chemical structure of BHX.

BHX, a small molecule synthesized in our laboratory, is a pyrazoline derivative. The chemical structure of BHX was depicted in Fig. 1. Previously, our studies showed that BHX exerted potent *in vitro* and *in vivo* anti-tumour activity against various solid tumours. BHX inhibited tumour cell proliferation, induced cell cycle arrest and apoptosis, decreased β-catenin protein levels, and increased E-cadherin expression^15^. However, the effects of BHX on breast cancer cells, and the specific molecules targeted by BHX responsible for its anti-tumour activity, are unknown. In this study, we describe the cytotoxic effects of BHX on MDA-MB-231 cells. We also investigate the anti-tumour effects and tolerability of BHX using a mouse xenograft model. The pharmacokinetics (PK) of BHX was measured by ultra-performance liquid-chromatography tandem mass spectrometry (UPLC-MS/MS). Finally, we attempt to elucidate the molecular pathways modulated by BHX that are necessary for its anti-tumour activity.

## Results

### *In vitro* cytotoxicity of BHX

**Figure 2:**
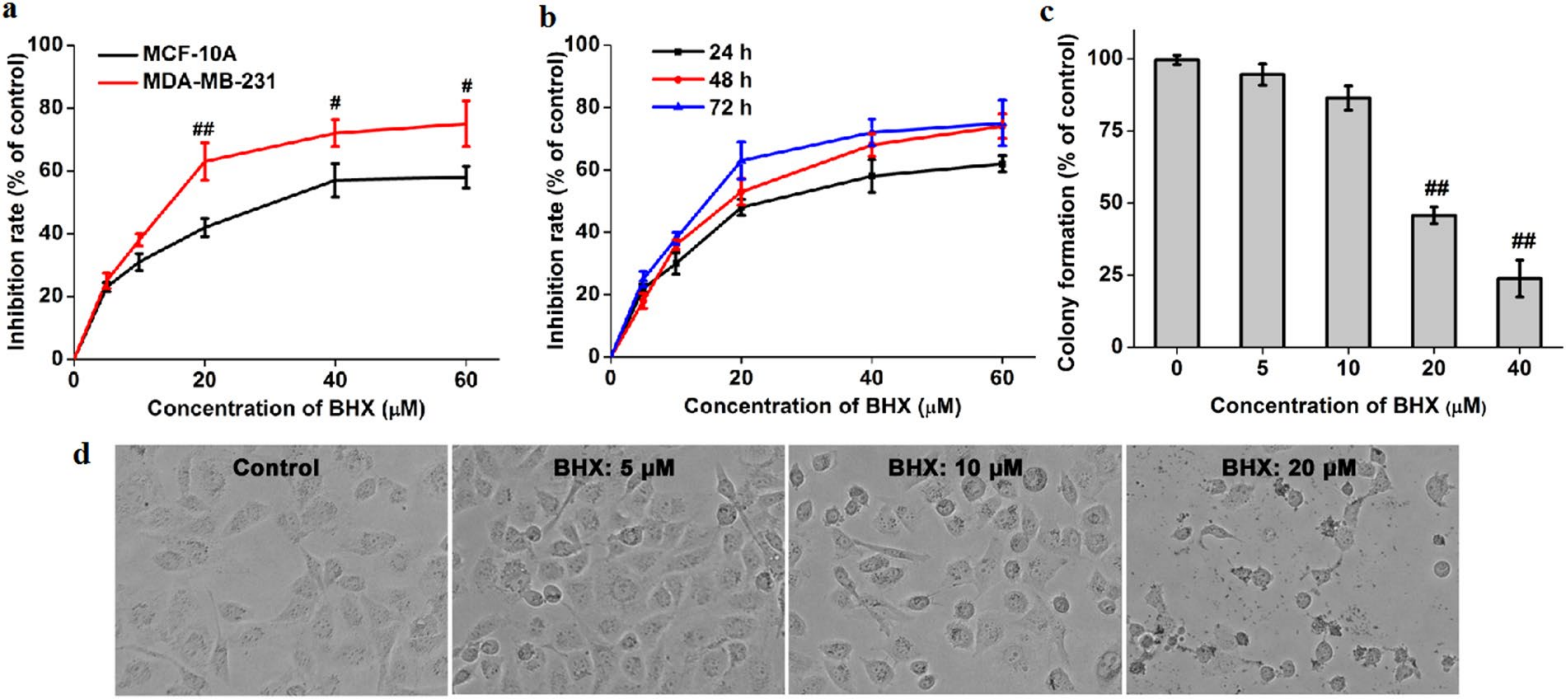
Effect of BHX on cell viability of MDA-MB-231 cells. (**a**) MCF-10A and MDA-MB-231 cells were treated with different concentrations of BHX for 72 h. (**b**) Cell viability was decreased in MDA-MB-231 cells at the indicated time points. **(c)** BHX suppresses colony formation of MDA-MB-231 cells. Results are expressed as means ± S.D. from three independent experiments. ^#^ *p* < 0.05 and ^##^ *p* < 0.01. **(d)** Morphological changes of MDA-MB-231 cells 24 h after incubation with BHX (200×). Black arrows indicate cell shrinkage and cell fragmentation.

To assess the effects of BHX on breast cancer cells, first we examined the cytotoxicity of BHX in human breast epithelial cell line MCF-10A (Fig. 2a). Result demonstrated that BHX displayed moderate cytotoxicity to the MCF-10A cells with an IC50 of 31.06 μM. Then we treated MDA-MB-231 cells with BHX (0–60 μM) for 24, 48, or 72 h. BHX exerted cytotoxic effects on MDA-MB-231 cells in a time- and concentration-dependent manner. Treatment with 20 μM for 24 h suppressed cell viability by 48.2 ± 2.6% (*p* < 0.05), and this effect was exacerbated at higher concentrations (40 and 60 μM, *p* < 0.01), with a maximal amount of suppression occurring at 72 h using a concentration of 60 μM (Fig. 2b). The IC50’s of BHX-induced suppression of cell viability for 72, 48, and 24 h were calculated to be 13.75, 19.28, and 28.06 μM, respectively. Additionally, BHX significantly suppressed the colony forming ability of MDA-MB-231 cells in a concentration-dependent manner, with an approximate IC50 of 20 μM (Fig. 2c).

### BHX alters the morphology of MDA-MB-231 cells

As shown in Fig. 2d, cells treated with BHX partially lost their epithelial morphology and displayed cell shrinkage and fragmentation. These morphological changes were more noticeable after incubation of MDA-MB-231 cells with relatively high concentrations of BHX.

### BHX suppresses the migration and invasion of MDA-MB-231 cells *in vitro*

**Figure 3:**
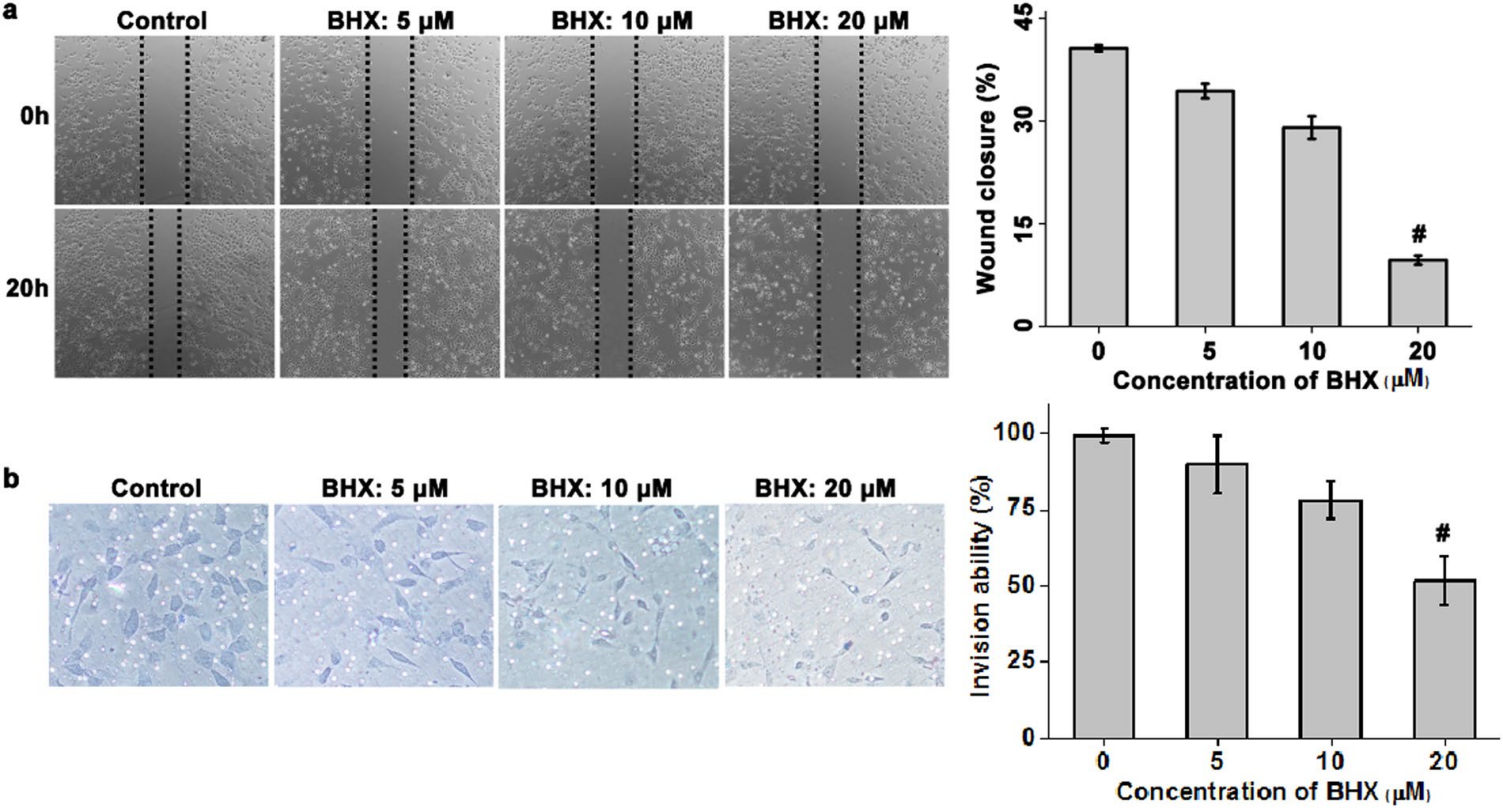
BHX suppresses EMT. Representative images and quantification of wound healing **(a)** and Transwell migration assay **(b)** of MDA-MB-231 cells after BHX treatment. Data are expressed as means ± S.D. from three independent experiments. ^#^ *p* < 0.05.

Using the scratch-wound assay (as shown in Fig. 3a), we found that treatment of MDA-MB-231 cells with BHX resulted in a significant reduction in cellular migration compared to the cells treated with vehicle only. BHX, at concentrations of 5, 10 and 20 μM, inhibited the migration of these cells by 34.38 ± 1.06%, 70.97 ± 1.65%, and 90.32 ± 0.68%, respectively. Using a Transwell assay, we observed a reduction in the number of migrating MDA-MB-231 cells in response to BHX treatment (Fig. 3b). After 24 h, BHX, at concentrations of 20 μM, significantly reduced the invasive potential of these cells by 48.38% (*p* < 0.05). In this assay, the number of cells that had invaded was normalized for effects on cell viability. Results indicated that cell migration and invasion were inhibited by BHX, regardless of the antiproliferative of the cells to BHX.

### BHX induces cell cycle arrest and apoptosis

**Figure 4:**
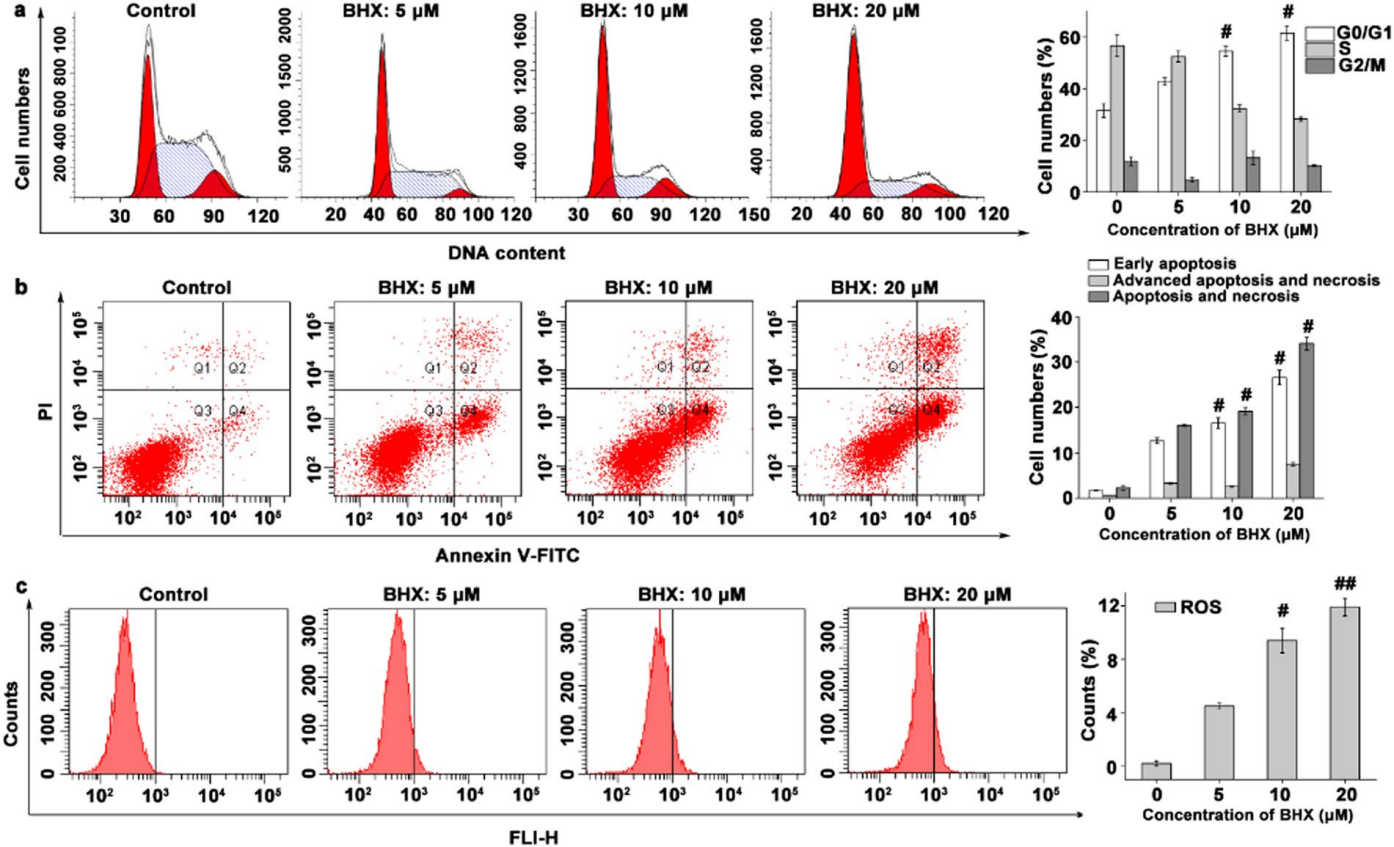
Effects of BHX on cell cycle progression, apoptosis, and intracellular ROS generation in MDA-MB-231 cells. BHX induced **(a)** G0/G1 arrest, **(b)** early apoptosis, and (**c)** intracellular ROS generation in MDA-MB-231 cells. Data are expressed as means ± S.D. from three independent experiments. ^#^ *p* < 0.05 and ^##^ *p* < 0.01.

We found that BHX increased the percentage of cells in the G0/G1 phase, with a recurrent decrease in the proportion of cells in the S and G2/M phases (Fig. 4a). After BHX treatment at concentrations of 10 or 20 μΜ, the percentage of MDA-MB-231 cells in the G0/G1 phase was found to be 54.49 ± 1.93% and 61.50 ± 2.85% (*p* < 0.05), respectively, whereas only 31.52 ± 2.67% of cells treated with vehicle were in the G0/G1 phase. Additionally, BHX increased the percentage of early apoptotic cells compared to cells treated with vehicle only (Fig. 4b).

### BHX promotes ROS generation and inhibits β-catenin in MDA-MB-231 cells

As shown in Fig. 4c, ROS generation was significantly increased in a dose-dependent manner in breast cancer cells exposed to BHX, with maximum potency observed at 20 μΜ. Western blotting showed that BHX suppressed β-catenin expression in a concentration-dependent manner. Moreover, BHX, at concentrations of 5, 10, and 20 μM, decreased β-catenin mRNA levels after 24 h of exposure by 4.61%, 25.73%, and 33.19%, respectively.

### BHX regulates EMT-related genes *in vitro*

**Figure 5:**
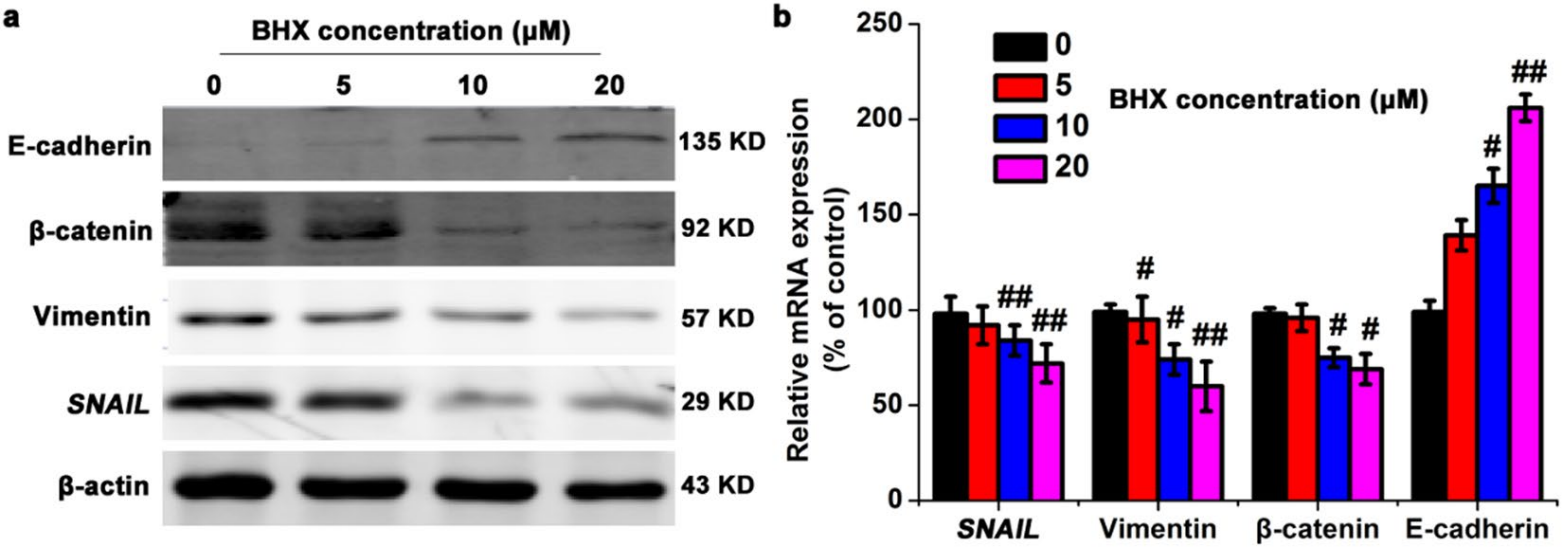
BHX alters the expression of proteins involved in EMT. **(a)** The effect of BHX on the protein expression levels of E-cadherin, β-catenin, Vimentin, and Snail, and β-actin was assessed by western blot. **(b)** The effect of BHX on the mRNA expression levels of E-cadherin, β-catenin, Vimentin, and Snail was assessed by PCR using GAPDH as a control. Data are represented as means ± S.D. from three independent experiments. ^#^ *p* < 0.05 and ^##^ *p* < 0.01.

We decided to investigate the effect of BHX treatment on the Wnt signalling pathway to elucidate the mechanism of anti-cancer activity of BHX. We found that treatment with BHX blocked EMT progression by up-regulating the protein and mRNA levels of E-cadherin and down-regulating Snail and vimentin expression in a dose-dependent manner (Fig. 5). After 24 h of exposure to 5, 10, and 20 μM of BHX, the mRNA levels of E-cadherin mRNA were significantly increased by 1.39-, 1.65-, and 2.06-fold, respectively, compared to the control. Snail expression was reduced by 7.03%, 15.88%, and 27.14% after treatment with 5, 10 and 20 μM BHX, respectively, while mRNA levels of vimentin were reduced by 4.89%, 26.27%, and 40.08%. Taken together, these results suggest that BHX regulates EMT in MDA-MB-231 cells.

### Anti-cancer effects of BHX *in vivo*

**Figure 6:**
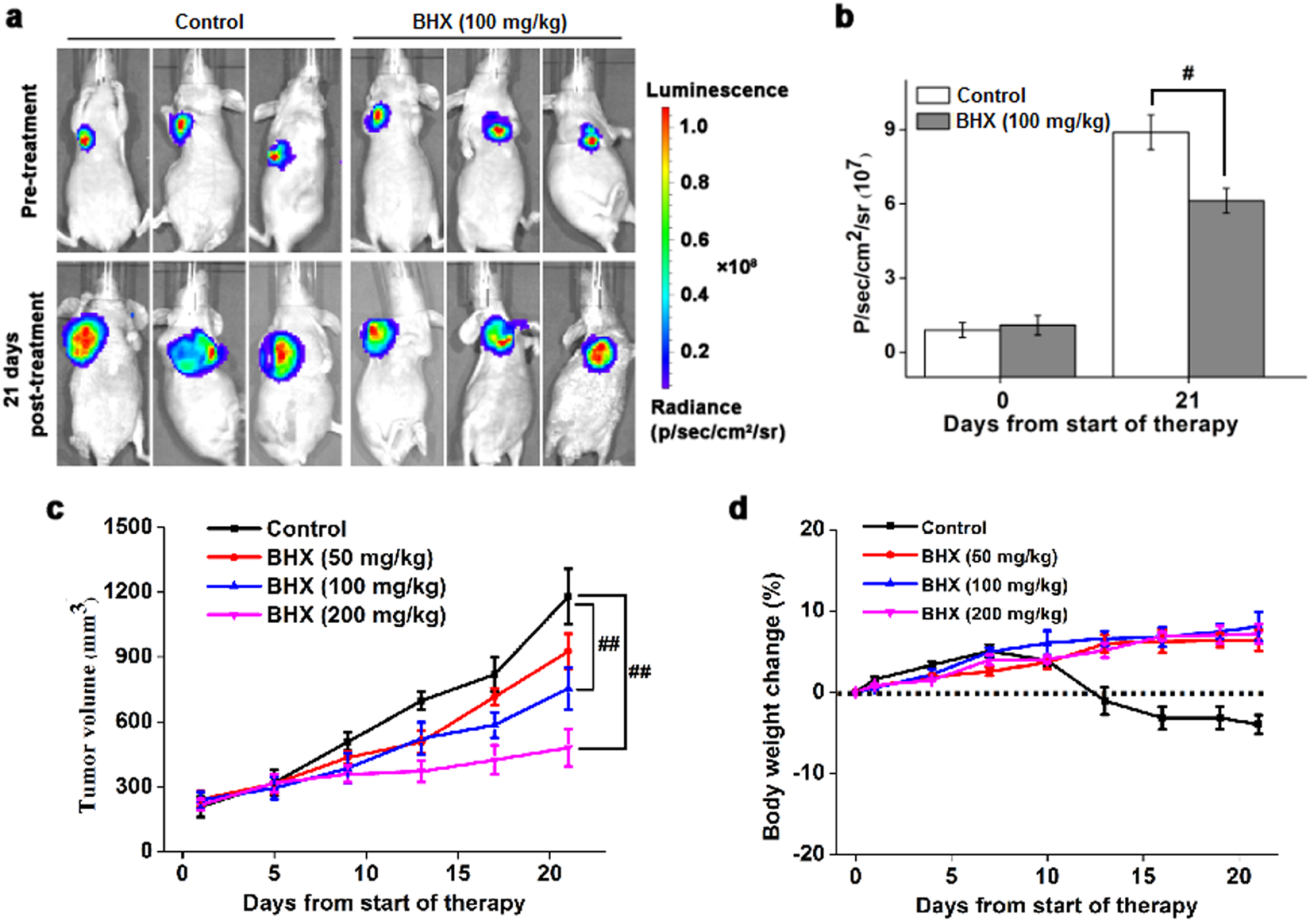
BHX reduces the growth of MDA-MB-231 tumours *in vivo*. **(a)** Bioluminescent imaging of MDA-MB-231 tumours at 0 and 21 days after treatment with BHX or vehicle. **(b)** Quantification of bioluminescent intensity in response to administration of BHX or vehicle after 0 and 21 days of treatment. **(c)** Tumour volume and **(d)** body weight were measured every 5 days after treatment with BHX or vehicle. Data are represented as means ± S.D. (n = 5). ^#^ *p* < 0.05 and ^##^ *p* < 0.01.

To assess the therapeutic effect of BHX on breast cancer growth *in vivo*, we developed a mouse xenograft model by subcutaneously transplanting MDA-MB-231 cells in BALB/c nude mice. Treatment with BHX (100, 200 mg/kg) resulted in a significant decrease in tumour size compared to the control group (*p* < 0.01). The average tumour volume of mice administered 100 mg/kg of BHX for 21 days was 754 ± 96 mm^3^, whereas tumour volume in mice fed vehicle only was found to be 1179 ± 128 mm^3^ (Fig. 6c). These data were confirmed as the bioluminescent intensity of tumours from mice administered BHX was significantly lower (6.13 ± 0.52 × 10^7^ p/sec/cm^2^/sr) compared to the control group (8.90 ± 0.74 × 10^7^ p/sec/cm^2^/sr, *p* < 0.05) (Fig. 6b). These data provide strong evidence that BHX significantly inhibits tumour growth *in vivo*. Moreover, we found no significant loss of body weight was observed in mice administered BHX (*p* > 0.05), indicating that there was no BHX-associated toxicity in these animals.

### *In vivo* pharmacokinetics

**Figure 7:**
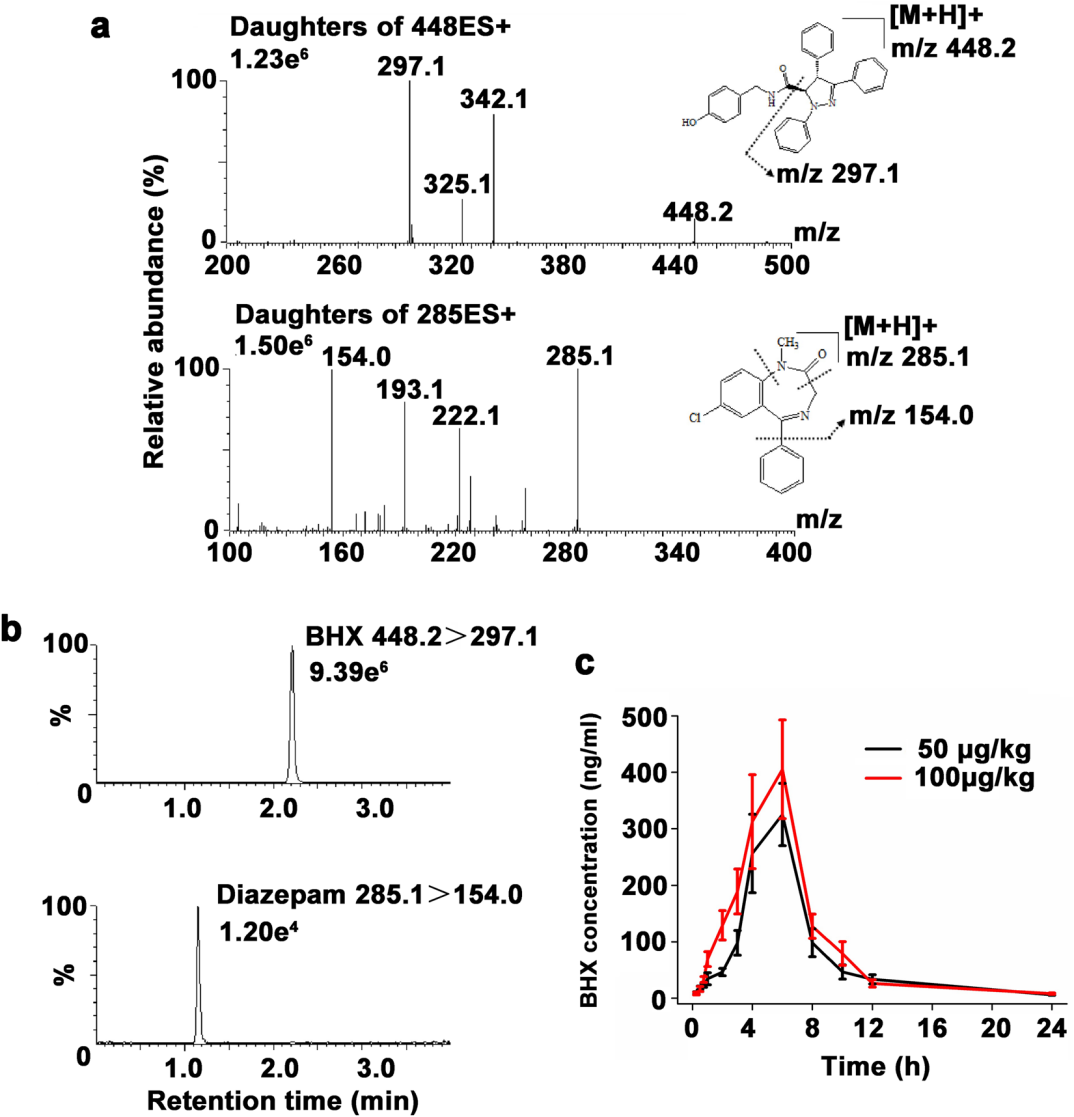
*In vivo* pharmacokinetics of BHX. **(a)** Electrospray ionization mass spectra of BHX and diazepam. **(b)** Multiple reactions monitoring (MRM) MS/MS chromatograms of BHX and diazepam. **(c)** Mean plasma concentration versus time plot followingadministration of BHX at concentrations of 50 and 100 μg/kg. Data were represented as means ± S.D. (n = 6).

Chromatographic separation was obtained with a mobile phase containing a high percentage of organic modifier, a low acid concentration, and with the fraction of acetonitrile raised to 90% during the separation process. Ionic strength and pH were stabilized by the addition of ammonium formate and formic acid to the mobile phase. A flow rate of 0.3 ml/min was used during the whole run without any loss in quality of the chromatographic separation. Figure 7b shows the MS spectra of BHX and diazepam. The optimized mass transitions of precursor to product ion/cone (V)/collision energies (V) for BHX and diazepam were 448.2 > 297.2/34/22 and 285.1 > 154.0/48/26, respectively. The retention times of BHX and diazepam were 2.2 min and 1.2 min, respectively.

The measurement of BHX concentrations in rat plasma by LC-MS/MS method was utilized in rats to investigate the time course of BHX after intraperitoneal injection. As illustrated in Fig. 7c, following administration, BHX plasma levels increased rapidly and reached the peak about 6 h post administration, and then decreased slowly. The PK parameters of BHX in rat plasma by non-compartmental analysis (NCA) are listed in Table 1. After BHX administration (50 μg/kg), the elimination half-life of BHX in plasma was 4.82 ± 0.93 min, the volume of distribution was 0.192 ± 0.047 L/kg and the clearance was 0.028 ± 0.006 L/h/kg.

**Table 1:** *In vivo* pharmacokinetics of BHX in rats following intraperitoneal injection at doses of 50 and 100 μg/kg.

Parameter	BHX 50 μg/kg	BHX 100 μg/kg
AUC_0-t_ (ng∙h/ml)	1773.76 ± 325.48	2300.01 ± 412.06
AUC_0-t_ (ng∙h/ml)	1816.25 ± 286.71	2347.91 ± 302.59
MRT_0-t_ (h)	6.80 ± 1.25	6.36 ± 1.32
t_1/2_ (h)	4.82 ± 0.93	4.11 ± 0.81
T_max_ (h)	5.37 ± 1.16	5.62 ± 1.38
CL (L/h/kg)	0.028 ± 0.006	0.043 ± 0.007
Vd (L/kg)	0.192 ± 0.047	0.253 ± 0.062
C_max_ (ng/ml)	325.15 ± 55.15	405.22 ± 87.52

Abbreviations: AUC, area under the concentration-time curve; MRT, mean residence time; t_1/2_, half-life; T_max_, time to maximum plasma concentration; CL, clearance; V_d_, volume of distribution; C_max_, maximum plasma concentration.

## Discussion

Aberrant activation of the Wnt/β-catenin signalling modulates a myriad of cellular processes such as proliferation, cell cycle disruption, migration, invasion, and apoptosis in a variety of malignancies^16^. Increased cell proliferation, migration, and invasion are widely considered as cancer hallmarks and key processes for tumour progression^17^. Therefore, inhibiting Wnt/β-catenin signalling pathway has the potential to delay breast cancer tumorigenesis and metastasis. In recent years, the pyrazoline family has attracted a great deal of attention due to its anti-tumour effects in various cancers, including breast cancer^18^. BHX, a novel low molecular weight pyrazoline derivative, was previously synthesized in our laboratory. We have demonstrated that BHX exhibits cytotoxic effects in multiple solid tumour cell lines, including A549, HT29, and MGC803^15^. In the present study, we have further verified that BHX suppresses not only cell viability but also cell migration and invasion, and induces cell cycle G1 phase arrest. We have also demonstrated that BHX suppresses the *in vitro* metastatic effect of MDA-MB-231 cells through inhibition of EMT, as BHX upregulated E-cadherin and down-regulated Snail, vimentin, and β-catenin.

Recently, EMT has become a major focus of translational cancer research, as molecular and morphologic features of EMT are found to correlate with poor histological differentiation, loss of tissue integrity, and metastasis^19^. During the process of EMT, cancer cells acquire mesenchymal characteristic, losing their epithelial features, while the rate of cell migration and invasiveness induced by cytokines is elevated^20^. Tumour cells adhere to the basal membrane and extracellular matrix (ECM), secreting numerous proteases that degrade the ECM and allow the cells to migrate through biological barriers^21^. Many previous studies have shown that the regulation of EMT represents an emerging therapeutic approach to treating breast cancer. Our results indicate that BHX suppresses the viability of MDA-MB-231 cells in a concentration- and time-dependent manner. Furthermore, the rates of invasion and migration of these cells were significantly decreased after BHX treatment. Interestingly, the Wnt pathway has been found to correlate with the invasion and proliferation of tumour cells^22^. In breast cancer tissues, β-catenin is aberrantly activated. Moreover, inhibition of the Wnt/β-catenin pathway reduces the epithelial-mesenchymal transition (EMT) and suppresses the invasion of breast cancer cells^23^. β-catenin also acts as an integral component of adherent junctions, helping maintain the epithelial characteristics of several types of cells, and participates in the Wnt signalling pathway as a downstream target^24^. During EMT, β-catenin mediates the binding of cadherins and cytoskeleton, and is a co-factor in a transcription factor coactivation complex comprised of β-catenin, TCF, and LEF, which directly regulates EMT-associated genes, including *SNAIL* ^25^. In normal epithelial cells, β-catenin is localized to the cell membrane; upon induction of EMT, it translocates to the cytoplasm or the nucleus and promotes the expression of EMT-related genes^26^. Snail promotes EMT through suppression of epithelia-associated proteins, like E-cadherin and claudins. Down-regulation of E-cadherin results in the destruction of intercellular connections and contributes to the metastasis of tumour cells^27^. Vimentin is up-regulated by Snail, and is considered to be a controversial marker of EMT. Studies have suggested that the expression of vimentin positively correlates with tumour metastasis. Therefore, vimentin is regarded as a critical marker of cancer-related EMT^28^.

In the present study, the protein and mRNA level of β-catenin, E-cadherin, vimentin, and Snail were assessed in MDA-MB-231 cells after BHX treatment. Our results confirm that BHX inhibits Wnt/β-catenin signalling pathway through degradation of β-catenin. We further show that BHX modulates the expression of E-cadherin by downregulating vimentin and Snail mRNA and protein expression. Finally, BHX effectively inhibited tumour growth in our xenograft breast cancer model. Therefore, additional studies are required to determine the exact molecular targets of BHX that contribute to its cytotoxic effects in breast cancer cells. Our findings provide a rationale for further preclinical evaluation of BHX in breast cancer management.

Taken together, we found that BHX modulated cell viability, migration, invasion, and cell cycle G1 phase arrest by down-regulating Wnt/β-catenin signalling and reversing EMT. BHX also slowed the growth of nude mice xenografts. Therefore, BHX functions as a tumour suppressing small molecule that can inhibit Wnt/β-catenin signalling and metastasis in breast cancer and may serve as a potential therapeutic agent in breast cancer treatment.

## Methods

### Materials

MDA-MB-231-luc cells were a kind gift from Professor Niu Ruifang (Tianjin Medical University Cancer Institute and Hospital). MCF-10A cell line was acquired from the American Type Culture Collection. MDA-MB-231-luc cells were cultured in Dulbecco’s Modified Eagle’s Medium (DMEM) supplemented with 10% foetal bovine serum (FBS), penicillin (100 units/ml), and streptomycin (100 μg/ml) at 37 °C with 5% CO_2_ in a humidified incubator. The medium for MCF-10A cells is composed of DMEM/F12 supplemented with 5% horse serum, 0.5 μg/ml hydrocortisone, 100 ng/ml cholera toxin, 10 μg/ml insulin and 20 ng/ml recombinant human epidermal growth factor (EGF). DMEM, FBS, F12, horse serum, hydrocortisone, cholera toxin, insulin, EGF, penicillin, and streptomycin were purchased from Gibco (Gaithersburg, MD). Antibodies raised against β-catenin, c-jun, c-myc, Cyclin D1, β-actin, Bax, and Bcl-2 were obtained from Cell Signaling Technology (Danvers, MA, USA). IRDye-conjugated anti-rabbit and anti-mouse IgG secondary antibodies were purchased from LI-COR Biosciences (Nebraska, USA). BCA protein assay kits were purchased from Thermo Fisher Scientific (USA). PVDF membranes were supplied by Millipore (Billerica, MA, USA). BHX was synthesized as previously described^15^.

### MTT assay

Cells were seeded in 96-well tissue culture plates for 24 h, then incubated with different concentrations of BHX for 24, 48, and 72 h. After incubation, MTT solution (5 mg/ml, 20 μl per well) was added and incubated for 4 h. The culture media were then replaced with dimethyl sulfoxide (DMSO, 150 μl). After shaking for 5 min, the intensity was measured at 490 nm using a SpectraMax PLUS 384 (Molecular Devices). The results are expressed as mean percentage of viable cells relative to untreated cells.

### Clonogenicity assay

To determine clonogenicity, MDA-MB-231 cells were plated in 6-well plates at a density of 1 × 10^3^ cells per well and exposed to different concentrations of BHX for 48 h. The culture media was then aspirated and fresh cell culture media was added to allow colony formation. The culture media was changed every third day. The colonies were fixed with ice-cold methanol for 5 min and stained with 1.0% crystal violet for 30 min. Colony numbers were counted and representative photos were taken.

### Cell cycle analysis

Cell cycle analysis was carried out as previously described^29^. The cells were harvested with trypsin 24 h after BHX treatment. They were washed thrice with ice-cold phosphate-buffered saline (PBS) and fixed with 75% ethanol at −20 °C overnight. The cells were then washed, incubated with 5 μl of RNase (0.25 mg/ml) at 37 °C for 30 min, pelleted, resuspended in propidium iodide (PI, 50 μg/ml), and incubated in the dark at room temperature for 15 min. Cell cycle analysis was analysed using a flow cytometer (FACSCanto II, BD, San Jose, CA).

### Detection of apoptosis

Flow cytometry was performed using PI and FITC-labelled annexin V (BD Pharmingen, CA, USA) staining according to the manufacturer’s protocol. Briefly, after 24 h incubation with BHX, cells were collected, resuspended in binding buffer (1×), and incubated with 5 μl of Annexin V and 5 μl of PI. After incubation at room temperature in the dark for 15 min, 400 μl of binding buffer (1×) was added. The samples were analysed within 1 h by flow cytometry (FACSCanto II, BD, San Jose, CA).

### Transwell and wound healing assays

Cell invasion and migration were measured using Transwell and wound healing assays, respectively. For Transwell assays, 2 × 10^4^ cells were suspended in serum-free media and seeded in the top chamber of Matrigel-coated invasion inserts (Corning life science, Corning, NY). BHX was then added to the top chambers. DMEM containing 10% FBS was added to the lower chamber as a chemoattractant. After 24 h incubation, the non-invading cells on the upper surface of the membrane were wiped away, and the invading cells on the lower surface of the membrane were fixed using 95% ethanol and stained with 0.1% crystal violet. Images were captured at ×200 magnification using an inverted phase contrast microscope (BX61, Olympus, Japan). In parallel, cells were treated identically in 24-well plate. These cells were harvested, stained with 0.1% crystal violet, and counted. The number of cells that had invaded was normalized for effects on cell viability. For wound healing assays, cells were seeded at 2 × 10^5^ cells/well in 24-well culture plates and allowed to reach confluence. The cell monolayers were scratched with a 200 μl sterile pipette tip to form wound gaps, washed thrice with PBS, and incubated with BHX. Images of the scratches were recorded at ×40 magnification using a phase contrast microscope (EVOS, AMG, USA) at 0 and 20 h. The areas between leading edges were measured using Image J software, after which the rates of wound healing were calculated.

### Measurement of intracellular ROS

The levels of intracellular ROS were monitored using dichlorofluorescein diacetate (H_2_DCFDA) as previously reported^30^. Briefly, MDA-MB-231 cells were incubated in 6-well plates (10^5^ cells/ml) for 24 h, and treated with BHX for 24 h. The culture media were replaced with 1 ml of H_2_DCFDA/RPMI DMEM media and incubated at 37 °C for 30 min, with a final H_2_DCFDA concentration of 10 μM. The levels of ROS were then detected using flow cytometry (FACSCanto II, BD, San Jose, CA).

### Western blotting

Total cell lysates were obtained by lysing the cells in SDS lysis buffer containing a protease inhibitor (PMSF), and protein concentrations were determined using a BCA protein assay kit. The proteins were separated by SDS-PAGE and transferred to PVDF membranes. The membranes were incubated with primary antibodies against Snail, Vimentin, β-catenin, and E-cadherin (1:1000 dilution) overnight at 4 °C, rinsed with TBST, and incubated with IRDye-conjugated anti-rabbit or anti-mouse IgG secondary antibodies (1:1000, LI-COR Biosciences). A β-actin-specific monoclonal antibody (1:1000 dilution) was used as a loading control. Bands were visualized with an Odyssey LI-COR infrared imaging system (LI-COR, Lincoln, NE).

### RNA isolation and PCR analysis

Total RNA from MDA-MB-231 cells was isolated using TRIzol (Life Technologies, USA). RNA samples were quantified using a NanoDrop 3300 spectrophotometer (Thermo Scientific, NanoDrop Products, Wilmington, DE, USA). Synthesis of cDNA was performed using a RevertAid First Strand cDNA Synthesis kit per manufacturer’s protocols (Thermo Scientific, USA). All primer sequences are listed in Table 2. The reaction mixtures were heated at 95 °C for 10 min, followed by 30 cycles at 94 °C for 30 s, 58 °C for 30 s, 72 °C for 20 s, and a final extension at 72 °C for 5 min. Subsequently, PCR products were electrophoresed in a 1.5% agarose gel and scanned using a gel/fluorescence image analysis system. GAPDH was selected as a loading control to standardize the amount of cDNA from each sample.

**Table 2:** Primers used for PCR analysis.

Gene	Primer sequence	
	Forward (5′-3′)	Reverse (5′-3′)
*SNAIL*	TAGCGAGTGGTTCTTCTGCG	GGGCTGCTGGAAGGTAAACT
Vimentin	GAGAACTTTGCCGTTGAAGC	GCTTCCTGTAGGTGGCAATC
β-catenin	GGCAGCAACAGTCTTA	GTCTCAGGGAACATAGC
E-cadherin	TGCCCAGAAAATGAAAAAGG	GTGTATGTGGCAATGCGTTC
GAPDH	GAAGGTGAAGGTCGGAGTC	GAAGATGGTGATGGGATTTC

### Animal experiments

The experimental design of the animal studies was approved by the Ethics Committee of the Tianjin Medical University Cancer Institute and Hospital. All animal studies were performed in accordance with protocols approved by the Institutional Animal Care and Use Committee of the Tianjin Medical University Cancer Institute and Hospital.

### Anti-tumour effect *in vivo*

Female BALB/c nude mice (18 ± 2 g) were purchased from Institute of Laboratory Animal Sciences, Peking Union Medical College (Beijing, China). MDA-MB-231 tumour bearing mice were established as described previously^31^. When the average tumour volume reached between 200 and 250 mm^3^, the mice were randomly assigned to either BHX- or vehicle-treated groups (five mice per group). BHX or vehicle was administered by daily intraperitoneal injection at doses of 50, 100 and 200 mg/kg for 21 consecutive days. The tumour length and width were measured using callipers and the tumour volume was calculated twice weekly. The body weight of mice was determined at least twice weekly during the study period.

For mice bearing MDA-MB-231-luc tumours, measurement of luminescence was conducted prior to administration, and 21 d post-administration of BHX or control using an *in vivo* IVIS spectrum imaging system (Caliper Life Sciences, Waltham, MA, USA) as previously described^31^. These data were analysed using Xenogen Living Image^®^ software.

### *In vivo* pharmacokinetics

Male Sprague-Dawley (SD) rats (250 ± 50 g body weight) were purchased from Institute of Laboratory Animal Sciences, Peking Union Medical College (Beijing, China). Rats were housed in a light-controlled room at 25 ± 2 °C and 55 ± 5% relative humidity, at the Animal Center of Tianjin Medical University Cancer Institute and Hospital (Tianjin, China). Animals received a standard diet and water *ad libitum*. Treatment with solutions of BHX was given at doses of 50 μg/kg or 100 μg/kg via intraperitoneal injection. At 0, 10, 20, 30, 40 min, 1, 2, 3, 4, 6, 8, 10, 12, and 24 h post injection, blood samples were collected into heparinized glass tubes by direct venipuncture. Plasma was prepared from the blood by centrifugation at 13,000 rpm for 10 min at 4 °C and stored at −80 °C until further analysis.

An ACQUITY™ ultra-performance liquid chromatography system connected to a Xevo triple quadrupole mass spectrometry system (Waters, MA, USA) equipped with an electrospray ionization (ESI) source was used in this study. Aliquots (50 μl) of plasma was vortex-mixed with methanol (400 μl) containing diazepam (internal standard, 200 ng/ml concentration) for 2 min and then centrifuged at 13,000 rpm at 4 °C for 10 min. Then, 400 μl of supernatant was transferred to a clean centrifuge tube and dried with an N_2_ gas stream. Samples were reconstituted with 100 μl methanol/H_2_O (50/50, v/v). The injection volume of the assay was 5 μl. The mobile phase (MP) was composed of MPA (acetonitrile) and MPB (5 mM ammonium formate with 0.1% formic acid in water), and the flow rate was maintained at 0.3 ml/min. BHX was separated on a BEH C-18 column (1.7 μm, 2.1 mm × 50 mm) (Waters, MA, USA) with the following MP gradient: MPA: 50% (0–0.01 min), from 50–90% (0.01–3.0 min), from 90–50% (3.0–3.2 min), and 50% (3.2–4.0 min). A switching valve directed the flow eluting between 1.0–2.5 min into a mass spectrometer, and the remainder to a waste container. BHX and diazepam were ionized with ESI in the positive mode and fragmented with collision gas for analysis using multiple reaction monitoring (MRM). The ESI source settings for the analysis were as follows: 350 °C gas temperature; 650 L/h desolvation; 50 L/h cone; 2.5 kV capillary voltage. The product ions were 448.2 > 297.2 for BHX and 285.1 > 154.0 for diazepam. The data acquisition and processing were done using MassLynx Mass Spectrometry software, version 4.1 (Waters, MA, USA). The dwell time was 400 ms for all ion transitions monitored.

The PK parameters of BHX, including the elimination half-life (t_1/2_), area under the curve for drug concentration from time zero to infinity (AUC_0-∞_), total body clearance (CL), volume of distribution (V_d_), and mean residence time (MRT), were calculated using the PK analysis system, DAS 2.1 (Anhui, China).

### Statistical analysis

Statistical analysis was performed using SPSS version 12.0 (SPSS Inc., Chicago, IL, USA). All values are expressed as means ± SD. Statistical differences were evaluated using a Student’s test for two groups and analysed by one-way ANOVA for multiple groups. A value of *p* < 0.05 was considered statistically significant.

### Data availability

All data generated during the current study are included in this article.
